# Identification of FDA-approved drugs against SARS-CoV-2 RNA-dependent RNA polymerase (RdRp) through computational virtual screening

**DOI:** 10.1007/s11224-022-02072-1

**Published:** 2022-11-25

**Authors:** Dhananjay Jade, Areej Alzahrani, William Critchley, Sreenivasan Ponnambalam, Michael A. Harrison

**Affiliations:** 1 School of Biomedical Sciences, University of Leeds, Leeds, UK; 2 School of Molecular & Cellular Biology, University of Leeds, Leeds, UK

**Keywords:** SARS-CoV-2, RNA-dependent RNA polymerase, FDA, Virtual screening, Docking, Molecular simulation

## Abstract

The SARS-CoV-2 coronavirus is responsible for the COVID-19 outbreak, which overwhelmed millions of people worldwide; hence, there is an urgency to identify appropriate antiviral drugs. This study focuses on screening compounds that inhibit RNA-dependent RNA-polymerase (RdRp) essential for RNA synthesis required for replication of positive-strand RNA viruses. Computational screening against RdRp using Food and Drug Administration (FDA)-approved drugs identified ten prominent compounds with binding energies of more than − 10.00 kcal/mol, each a potential inhibitor of RdRp. These compounds’ binding energy is comparable to known RdRp inhibitors remdesivir (IC50 = 10.09 μM, SI = 4.96) and molnupiravir (EC50 = 0.67 − 2.66 µM) and 0.32–2.03 µM). Remdesivir and molnupiravir have been tested in clinical trial and remain authorized for emergency use in the treatment of COVID-19. In docking simulations, selected compounds are bound to the substrate-binding pocket of RdRp and showed hydrophobic and hydrogen bond interaction. For molecular dynamics simulation, capmatinib, pralsetinib, ponatinib, and tedizolid phosphate were selected from the initial ten candidate compounds. MD simulation indicated that these compounds are stable at 50-ns MD simulation when bound to RdRp protein. The screen hit compounds, remdesivir, molnupiravir, and GS-441524, are bound in the substrate binding pocket with good binding-free energy. As a consequence, capmatinib, pralsetinib, ponatinib, and tedizolid phosphate are potential new inhibitors of RdRp protein with potential of limiting COVID-19 infection by blocking RNA synthesis.

**Supplementary Information:**

The online version contains supplementary material available at 10.1007/s11224-022-02072-1.

## Introduction

The global pandemic of SARS-CoV-2 has been a global health emergency caused by the 2019 novel coronavirus (COVID-19). As of 7 July 2021, it has infected over 184.32 million globally, with 3.99 million deaths [1]. This COVID-19, that caused a pneumonia outbreak and caught attention worldwide in December 2019, is a newly identified β-coronavirus, first reported in Wuhan, China [2–5]. It has subsequently spread across 230 countries in a growing pandemic that has developed into a global health emergency. The World Health Organization (WHO) declared COVID-19 a global pandemic on 11 March 2020 [6, 7]. In 2002, a coronavirus outbreak in China caused a fatal respiratory illness and hence was referred to as severe acute respiratory syndrome coronavirus (SARS-CoV) [8, 9]. SARS-CoV killed around 750 people [10]. In 2012, another coronavirus outbreak in the human population of the Middle East, called Middle East respiratory syndrome (MERS), caused similar severe respiratory symptoms [11]. MERS-CoV killed around 866 people [12]. SARS-CoV-2 is reported to be more infectious than MERS-CoV or SARS-CoV [13].

The novel 2019 coronavirus SARS-CoV-2 belongs to the β-coronavirus (β-CoV) family, mainly infecting the gastrointestinal and respiratory tract. Coronavirus particles contain crown-like spikes on the surface, which can interact with the angiotensin-converting enzyme isoform 2 (ACE2) to facilitate infection [14–18]. The mortality rate for SARS-CoV-2 is 2.3%, considered to be lower in comparison with MERS (34.4%) and SARS (9.6%). However, SARS-CoV-2 does more rapidly infect and has caused greater mortality in a short period [10, 12, 13]. SARS-CoV-2 transmission from person to person, asymptomatic transmission, and prolonged symptomatic development substantially increase mortality in the older population [19–21]. Anti-coronaviral drug therapy approaches aim to inhibit viral RNA synthesis and hence virus replication, block the virus from interacting with human cell receptors, or restrain the virus self-assembly processes [22–24].

**Fig. 1:**
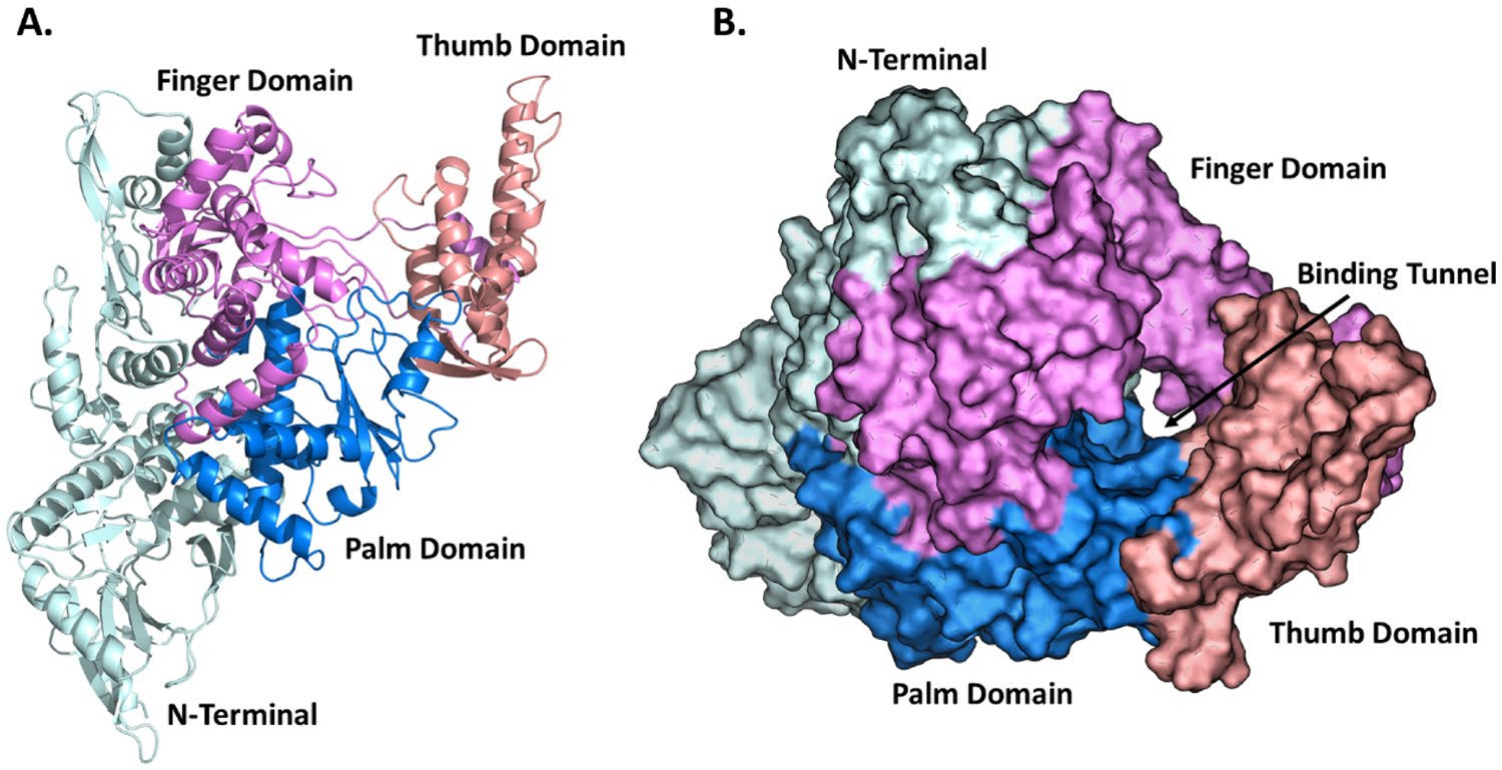
Structural model of SARS-CoV-2 RNA-dependent RNA polymerase. **A** RdRp protein structure in the right-handed form with three domains highlighted: finger domain (violet), palm domain (blue), and thumb domain (pink). The N-terminal region of RdRp is also shown (cyan). **B** Space fill model highlighting the RNA template binding tunnel/active site presence between three domains. Colouring scheme as in **A**

Coronavirus belongs to the *Coronaviridae* family, which is sub-classified into alpha (α), beta (β), gamma (ɣ), and delta (δ) coronavirus [24, 25]. Among these subclasses, alpha (α) and beta (β) types are responsible for infections in mammals. Gamma (ɣ) and delta (δ) cause infection in birds [26]. The SARS-CoV-2 genome is 29.8 kb in size and belongs to the genus β-coronavirus. It encodes four structural proteins, Spike (S), Envelope (E), membrane (M), and Nucleocapsid (N), and 16 non-structural proteins (NSPs), NSP1-10 (ORF1a) and NSP12-16 (ORF1b) [27–30]. Nsp12 is the RNA-dependent RNA polymerase (RdRp) enzyme that carries out RNA synthesis in all positive-strand RNA virus replication processes [31, 32]. Two Zn^2+^ ions are present in the RdRp structure, and these ions play a critical role in the stability of the RdRp tertiary structure. RdRp supports RNA synthesis by catalysing the RNA-template-dependent formation of phosphodiester bonds [30, 31, 33–37]. All the important functional sites are highly conserved among coronaviruses, including SARS-CoV-2 (Fig. 1). These include sites for template entry and binding, the polymerase reaction site (palm and finger domain comprising residues 398–814), and the product exit site through the tunnel (thumb) present in the residue 815–919 region [38, 39]. Studies examining the roles of NSPs in coronavirus replication have underlined the importance of RdRp, demonstrating that it makes an attractive potential target for anti-coronavirus drug design. Targeting NSP12 (RdRp) may therefore lead to potential treatment for COVID-19 [40, 41].

Coronavirus is one of the few RNA viruses to have a genomic regulation mechanism. Consequently, identifying nucleoside analogues that inhibit SARS-CoV-2 RNA replication has been difficult due to its unique exoribonuclease (ExoN) activity, which corrects errors in the growing RNA chain [42, 43]. Generally, the rate-limiting step for activating nucleoside analogues is the production of the nucleoside monophosphate. Nucleoside phosphoramidites such as remdesivir, favipiravir, and ribavirin are bioisosteres of monophosphates and bypass this rate-limiting step. Studies of FDA-approved compounds with antiviral activity have shown that efficacy can be highly variable and is dependent on the cell line used in the study. Tetrandrine, berbamine hydrochloride, abemaciclib, cepharanthine, and chloroquine showed four-fold higher IC50 values in a SARS-CoV-2-infected Calu-3 human lung carcinoma cell model, compared to a Vero primate epithelial model, whereas remdesivir, camostat mesylate, nafamostat mesylate, and cyclosporine show lower IC50 in Calu-3 cells [44]. For example, the IC50 for remdesivir in Vero cells is 11.41 µM, but in Calu-3 cells is 1.3 µM, which shows the 0.11 fold change. Nafamostat mesylate shows IC50 values of 13.88 µM and 0.0022 µM IC 50 in Vero and Calu-3 cells, respectively, and fold change is 0.00016 [44]. Hence, it is clear that quantitation of antiviral drug efficacy is highly dependent on the cell model being used.

Repurposing FDA-approved drugs is a fast-track option to identify new inhibitors of essential SARS-CoV-2 protein functions. At the initial stage of the pandemic, the WHO launched a trial treatment against COVID-19 using remdesivir, lopinavir plus ritonavir, chloroquine, and Interferon-β [45]. Lopinavir plus ritonavir and chloroquine were subsequently removed from the list of potential therapeutics because of uncertainty over benefits and possible risks of side effects, although they are still being actively investigated. To date, remdesivir (Veklury) is the only FDA-approved compound for the treatment of COVID-19 [46]. Remdesivir is a single Sp isomer of 2-ethylbutyl L-alanine phosphoramidate pro-drug that inhibits RNA synthesis and hence viral replication [47, 48]. The efficacy of chain-terminating nucleotide analogues requires viral RdRp to recognize and successfully incorporate the active form of the inhibitors into the growing RNA strands. Remdesivir diphosphate binding to COVID-19 virus RdRp/NSP12 has been modelled by superpositioning with sofosbuvir bound to HCV NS5b. Remdesivir is a metabolically active form that works by inhibiting viral RNA-dependent RNA-polymerase even with proofreading by viral exoribonuclease, which facilitates premature termination of viral RNA. Molnupiravir, which also inhibits RdRp, is a biological pro-drug of β-D-N(4)-hydroxycytidine (NHC), a nucleoside analogue with antiviral activity against SARS-CoV, SARS-CoV-2, MERS-CoV, respiratory syncytial virus, influenza virus, hepatitis C virus, bovine viral diarrhoea virus, and Ebola virus [47, 49]. Molnupiravir has shown beneficial effects in mildly and moderately symptomatic COVID-19 patients. Remdesivir, being a pro-drug, is metabolized into its active form, GS-441524 [50]. This metabolite, which is the predominant metabolite of remdesivir in plasma, is an effective inhibitor of RNA replication of SARS-CoV-2 in Vero E6 and other cells.

The aim of this study was to conduct an initial virtual screen of FDA-approved compounds in the DrugBank database, using 2D similarity screening to identify molecules with similar characteristics to remdesivir and molnupiravir. Subsequently, molecular docking was used to narrow the search to compounds with binding interactions comparable to remdesivir and molnupiravir. We then selected the most promising hit compounds to check the stability of protein–ligand interaction through molecular dynamic (MD) simulation.

## Material and methods

### FDA-approved compound preparation

FDA-approved compounds for high-throughput virtual screening against RdRp protein were downloaded from DrugBank (https://go.drugbank.com/). The compounds were converted to 3D and mol2 file format by adding hydrogen molecules through the molconvert tool of InstJChem, ChemAxon software (http://www.chemaxon.com). Remdesivir [51] was used as a reference compound against RdRp.

### 2D similarity screening

The similarity between the reference compound remdesivir and FDA-approved compounds was calculated using the ScreenMD programs of InstJChem software (ChemAxon) [52]. Tanimoto coefficients (Tc) to quantify dissimilarity between reference and FDA-approved compounds were generated and ranked according to the Tc value. The threshold for Tanimoto coefficients (Tc) was set at ≥ 0.50. The dissimilarity score was then converted to a compound similarity score by subtraction (1-dissimilarity). The Tc molecular descriptor is a set of values associated with the compound’s structure. In this Tc calculation, we used a 2D fingerprint-based similarity search, the fastest and most robust compound-based approach, to screen the compounds from the multi-million compound database.

### Preparation of the RdRp structural model

The RdRp 2.95 Å resolution structural model determined by cryo-electron microscopy was downloaded from Protein Data Bank (PDB) (PDB id: 7BTF). RdRp RNA polymerase is a complex of three subunits: Non-Structural Protein 7 (NSP7, chain-C), Non-Structural Protein 8 (NSP8, chain-B and D), RNA-directed RNA-polymerase (NSP12, chain-A), and zinc ions (Zn^2+^) [53]. From the structural model, we removed water molecules, Zn^2+^, NSP7, and NSP8. The protein structure containing NSP12 (chain-A; Fig. 1) was prepared by adding missing unresolved residues using Swiss Model [54]. However, some residues from the N-terminal and C-terminal regions remained unresolved, but did not affect binding interactions because of their distance from the active site. After that, we added hydrogen atoms and subjected them to energy minimization using UCSF Chimera [55]. For MD simulation, two Zn^2+^ ions that contribute to RdRp were added to the structural model using MODELLER [56].

### Prediction of the active binding site

Residues involved in the RdRp active site were identified by literature survey [57]. Along with this, we cross-verified predictions of the binding site using the COACH meta-server [58]. Predicted residues and residues identified from the literature [57] were used to perform molecular docking.

### Molecular docking

Molecular docking was performed using AutoDock tools for the reference compounds and screen hit compounds. For docking purposes, the 3D formats of the reference compound and lead compounds were prepared by adding hydrogen bonds using the ‘Molconvert’ tool in ChemAxon software. AutoDock Tools (ADT) 1.5.6 [59] was used for the RdRp structure preparation, in which we added polar hydrogen and charges by Kollman charges methods [60]. The grid points 102 (x) × 108 (y) × 112 (z) and grid centre point 174.056 × 180.193 × 210.798 with a spacing of 0.375 Å were assigned to the protein. We kept all other docking parameters for this study as a default value. Grid maps were calculated using Autogrid4, and docking was performed using Autodock4 [61]. A total of 10 conformations were generated and sorted according to binding energy. Compounds that show the highest binding energy were selected for further study. Interactions between the selected compounds and RdRp were checked using protein–ligand interaction profile (PLIP) [62].

### Clustering

After molecular docking, we performed clustering for the selected hit compounds using the online ChemBioServer [63], based on the hierarchical clustering methods. Clustering was performed using the Clustering Linkage Selection-Ward linkage clustering method and Distance Selection-Soergel Distance method. For this clustering method, we used the 166-bit Open Babel MACCS fingerprint to generate the compound fingerprint. Through this hierarchical clustering approach, data were analysed iteratively, such that at each step, a pair of similar clusters were merged or a large cluster divided. This gives the ability to analyse large heterogeneous datasets. We identified homogeneous subsets from the heterogeneous datasets based on the similarity measures [64]. Selected compounds were compared with the reference compounds to check the difference in structure using ProFit server [65]. The most basic ProFits function is the superimposition of two ligand structures with provision for entering the zones over which the fitting and RMSD calculation is performed. The compounds that fell into the top ranked cluster were selected for further study by molecular dynamics simulation.

### MD simulation

Reference and screen hit compounds were used for 50-ns MD simulation using GROMACS (Version-5.1.4) [66]. GROMOS 53A6 force fields were used to generate the topology of protein [67]. The binding orientation of hit compounds was obtained after from the docking approach described above. The topology for the selected hit compounds was created using the PRODRUG online tool [68]. The simple point charge (SPC216) water molecules were used in solving the RdRp-hit compound complexes. All systems were neutralized by Na^+^ or Cl^−^ ions and energy minimization performed to relax the overall system. Temperature and pressure were stabilized with NVT and NPT. After 50-ns simulation, we calculate the root mean square fluctuation (RMSF) (g_rmsf), root mean square deviation (RMSD) (g_rms), and radius of gyration (Rg) (g_gyrate). Finally, we calculated the hydrogen bonds formed between compound and RdRp and protein solvation.

### Binding-free energy calculation

BFE plays a significant role in drug discovery, giving a quantitative estimation of the ligands binding to the protein. After completion of the MD simulation, we used the stable region of the RdRp-compound complex to calculate the binding-free energy (BFE), essential for studying the reciprocal recognition and binding of protein and ligands. The binding-free energy value is an accurate standard for evaluating the bending degree of proteins to accommodate ligands [69, 70]. The binding-free energy for selected hit compounds was calculated using molecular mechanics energies combined with Poisson-Boltzmann (MM-PBSA) method using the g_mmpbsa tool [71]. We used the g_mmpbsa module in GROMACS for energy change under vacuum conditions, calculated using molecular mechanics (MM) methods. PB shows the polar part of solvent-free energy of systems calculated by the Poisson-Boltzmann equation. The non-polar part of solvent-free energy systems is fitted by the solvent-accessible surface area (SASA).

$$\Delta {\varvec{G}}{\varvec{b}}{\varvec{i}}{\varvec{n}}{\varvec{d}}=\Delta {\varvec{E}}{\varvec{v}}{\varvec{d}}{\varvec{W}}+\Delta {\varvec{E}}{\varvec{e}}{\varvec{l}}{\varvec{e}}+\Delta {\varvec{G}}{\varvec{p}}{\varvec{o}}{\varvec{l}}+\Delta {\varvec{G}}{\varvec{n}}{\varvec{o}}{\varvec{n}}{\varvec{p}}{\varvec{o}}{\varvec{l}}-{\varvec{T}}\Delta {\varvec{S}}$$

where ΔEele and ΔEvdW are electrostatic and Van der Waals components, respectively. ΔGpol and ΔGnonpol are polar and non-polar components, respectively. TΔS is the temperature and entropic contribution toward binding-free energy (BFE).

### Tools and software used for data analysis

The RdRp-ligand complex interaction was visualized through PyMOL (https://pymol.org/2/). The 2D plot of RMSD, RMSF, Rg, RdRp-solvent hydrogen bond interaction, and RdRp-ligand hydrogen bond interaction of MD simulation graph was generated by Xmgrace (https://plasma-gate.weizmann.ac.il/Grace/).

## Results and discussion

### Compound screening using 2D similarity search

A total of 2509 compounds approved by FDA were downloaded from DrugBank in SDF format (https://go.drugbank.com/) and converted to 3D mol2 format by adding hydrogen. All compounds were converted in 3D mol2 format without error, ready for high-throughput virtual screening against RdRp. These 2509 compounds were used for the 2D similarity search, with remdesivir drug as a reference (query) compound. This reduced the library to 1299 compounds with Tc values ≥ 0.50 (Fig. s1). From this set of compounds, we selected the compounds with Tc values ≥ 0.8. There were 269 compounds within the score range 0.8–0.9, with a further 51 in the Tc range 0.9–1 score range. This average chemical fingerprint (CF) Tanimoto (Tan) score ranges between 0 and 1 Tc, with a higher Tc score indicating that the compound is more similar to the reference compound and vice versa. We selected 320 compounds applying the criterion of Tc value ≥ 0.80 (Fig. s1), and the selected compounds were used for molecular docking.

### Preparation of the RdRp structural model and validation

The three-dimensional co-ordinates of the RdRp structural model were downloaded from PDB (PDB ID: 7BTF). After modelling missing residues, we analysed the protein structure by Ramachandran plot (Fig. s2A). RdRp consists of a total of 928 amino acids. Of these, 760 residues (89.5%) are in the most favoured region, 82 residues (9.7%) are present in the additional allowed region, five amino acids (0.6%) fall in a generously allowed region, and two (0.2%) residues belong to the disallowed region (Fig. s2A). The modelled protein was energy minimized after addition of hydrogen atoms using the steepest descent steps = 100, steepest descent step size 0.02 Å, conjugate gradient steps = 10, and conjugate gradient steps size of 0.02 Å. The final energy minimized structure is shown in Fig. s2B. Further analysis of the modelled protein using Verify3D to determine the compatibility of the atomic model based on the location and environment showed that modelled protein passed the quality criteria with 88.12% of residues having an average 3D-1D score of 0.2 or better.

### Finding active binding sites

Active site residues in RdRp were identified through an analysis of the literature and cross-verified the binding site using the COACH meta-server. Residues involved in the substrate/template binding tunnel are V588, I589, G590, K593, W598, M601, G616, W617, D618, Y619, C622, S681, S682, G683, D684, A685, T686, T687, A688, Y689, N691, N695, M755, I757, L758, S759, D760, D761, A762, V763, K798, W800, E811, F812, C813, S814, Q815, and P830. This binding tunnel includes elements of the finger, thumb, and palm domains (Fig. 1B). The binding residues identified from the literature survey were also predicted using the COACH meta-server. We selected the top two consensus binding residues predicted by the COACH server, which show C-scores of 0.08 (1st site) and 0.06 (2nd site). This C-score is the confidence score of the prediction, ranging from 0 to 1 with a higher score indicating a more reliable prediction.

### Molecular docking conformation analysis

**Fig. 2:**
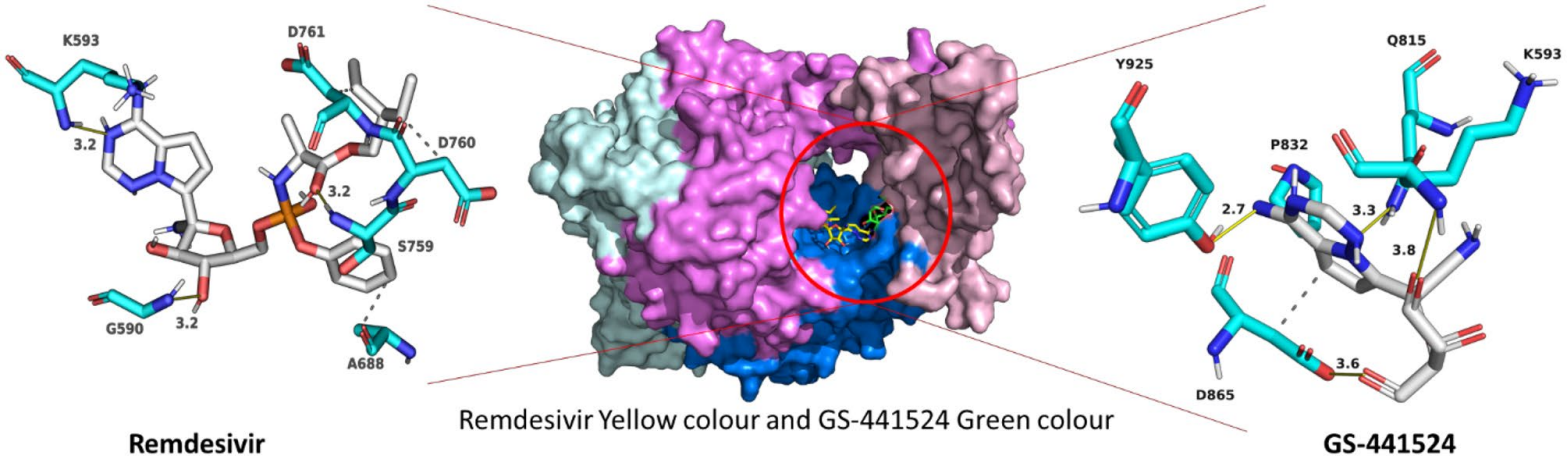
Remdesivir and GS-441524 binding to SARS-CoV-2 RdRP. Central image: Remdesivir (yellow) and its primary metabolite GS-441524 (green) bind to the mouth of the binding tunnel formed at the junction of the finger, palm, and thumb domains (colour scheme as in Fig. 1). Left and right images: Remdesivir and GS-441524 (white) form hydrophobic interactions and hydrogen bonds with residues of RdRp (cyan)

The 320 FDA-approved compounds identified by 2D similarity to remdesivir were used for molecular docking using AutoDock. Before that, we added hydrogen atoms to the RdRp protein, and the RdRp protein was neutralized by adding gasteiger charge molecules. The binding energies (BE) for these compounds were calculated, and the best performing compounds were selected on the basis of a BE cut-off value of − 10.00 kcal/mol (Table 1). For comparison, the reference compound remdesivir shows a binding energy of − 3.84 kcal/mol. Remdesivir forms hydrogen bond interactions with RdRp via residues G590, K593, and S759 with a distance of 3.2 Å (Table s1). There are also hydrophobic interactions with A688, D760, and D761 (Table s1). The active metabolite GS-441524 forms hydrogen bonds with residues K593 (3.8 Å), Q815 (3.3 Å), D865 (3.6 Å), and Y925 (2.7 Å) and hydrophobic interactions with P832 and D865. Both remdesivir and GS-441524 bind into the substrate/template binding tunnel (Fig. 2).

**Table 1:**
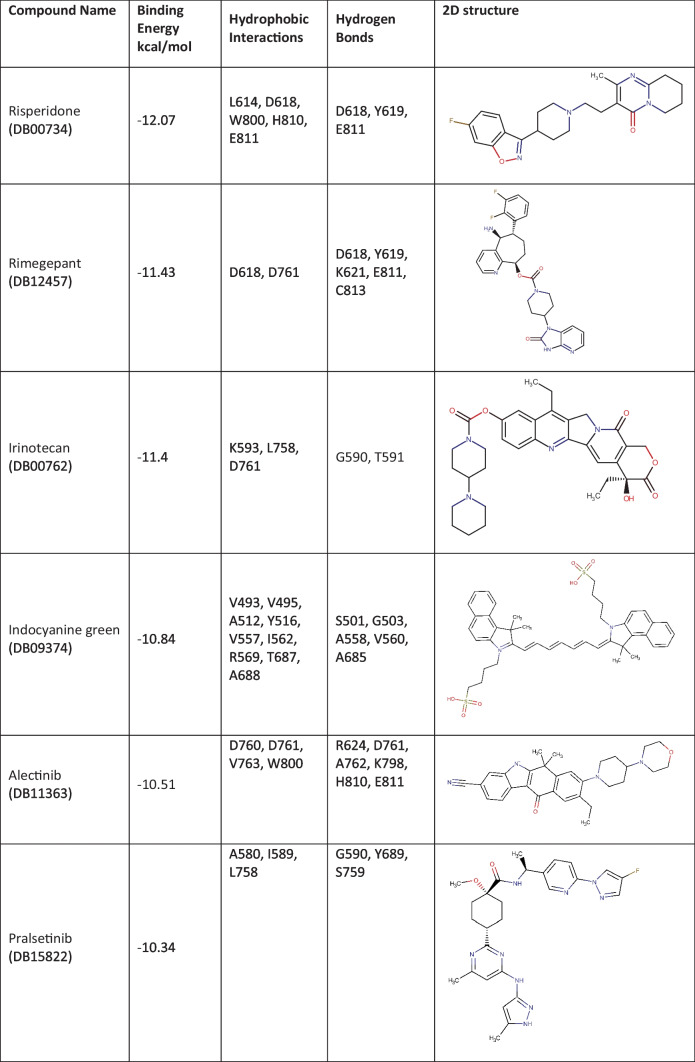

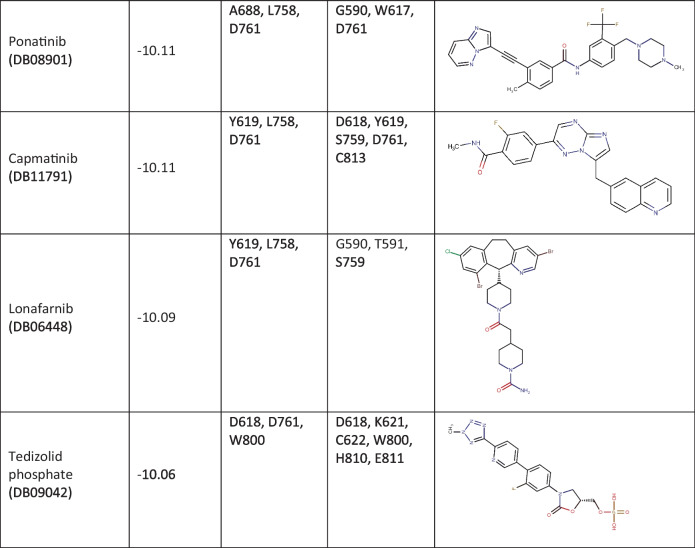
Binding characteristics and 2D structures of putative RdRp- binding compounds

**Fig. 3:**
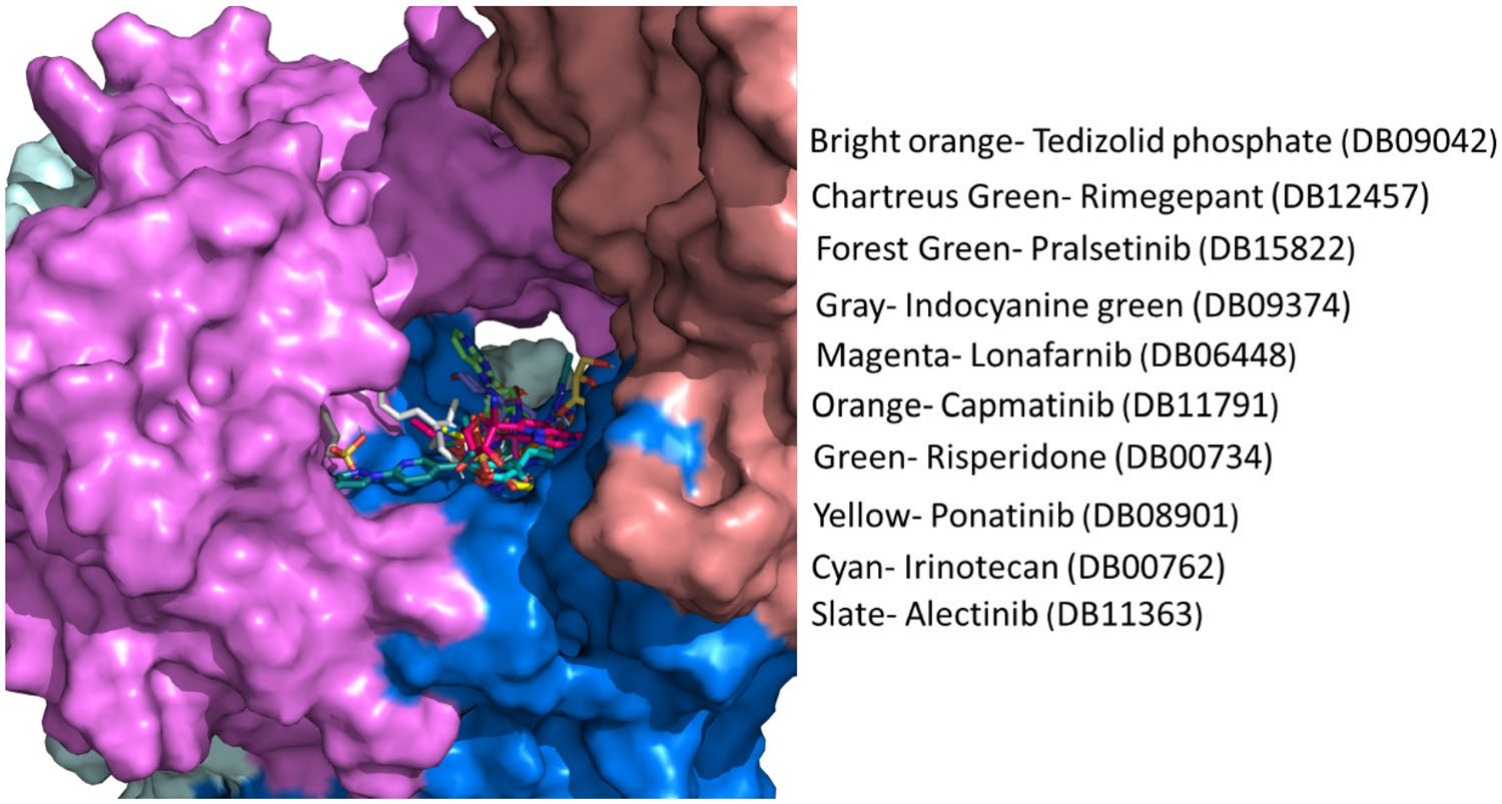
Binding of virtual screen hit compounds to SARS-CoV-2 RdRp. Ensemble binding of the ten identified hit compounds from the virtual screen is shown docked to the binding tunnel as for the reference compounds in Fig. 2. RdRp colour scheme as in Fig. 1. There is a high degree of overlap in the putative binding sites for the compounds

The 320 lead compounds were also docked into the binding tunnel. The top ten compounds with the lowest binding energy were selected (Table 1) and subsequently docked into the same position as remdesivir (Fig. 3). Previous studies have shown that some compounds from the list of hits do interact with RdRp [72–77]. Among the selected ten compounds, risperidone (DB00734) shows the lowest binding energy of − 12.07 kcal/mol, and the highest binding energy is shown by tedizolid phosphate (DB09042), the binding energy of which is − 10.06 kcal/mol. All ten docked compounds docked in the binding tunnel with significant overlap (Fig. 3).

**Fig. 4:**
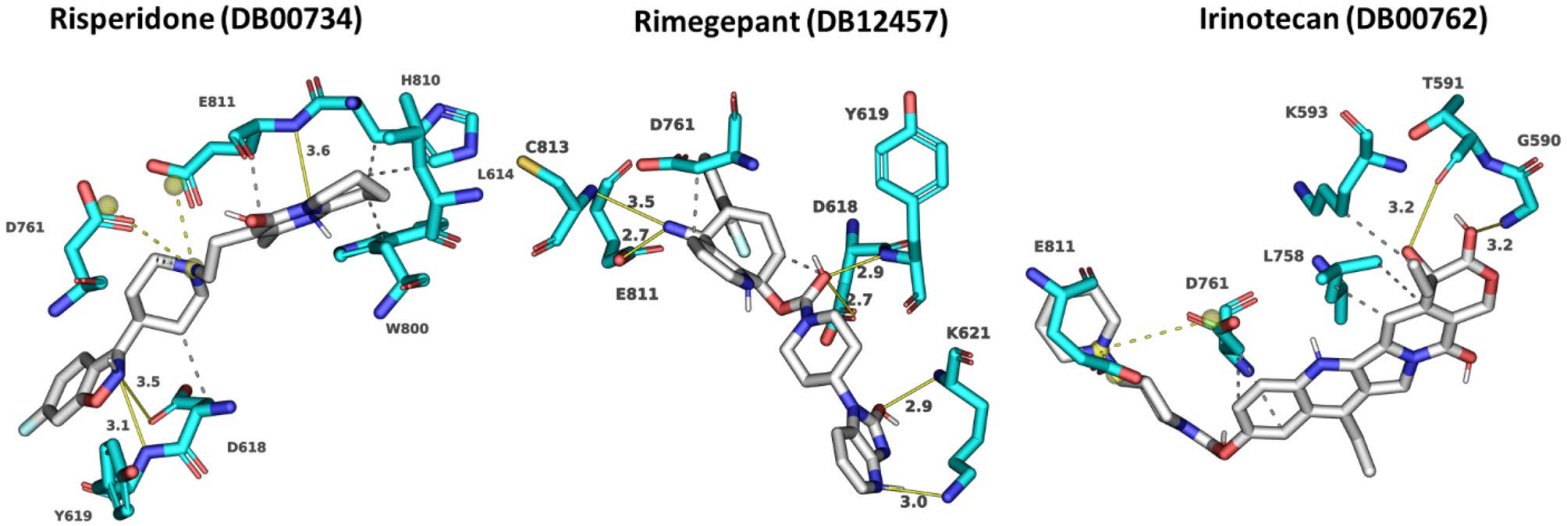
Modelled interactions between SARS-CoV-2 RdRp and risperidone, rimegepant, and irinotecan. Protein–ligand interactions formed between the screen hit compounds (grey) by hydrophobic interaction (grey dotted line), salt bridges (dotted yellow line), and H-bonds (solid yellow line) with numbered residues (cyan) in the binding tunnel of RdRp are shown

Risperidone (DB00734) shows the lowest binding energy, which forms putative hydrophobic interactions with L614, D618, W800, H810, and E811 and H-bonds at D618 (3.5 Å), Y619 (3.1 Å), and E811 (3.6 Å) (Table s1). This compound also forms salt bridges at D761 (5.0 Å) and E811 (3.4 Å) (Fig. 4). Risperidone is a second-generation anti-psychotic medication used to treat mental health disorders such as bipolar mania, schizophrenia, and psychosis or as an adjunct in severe depression [52, 78]. Compared with remdesivir, it shows a 0.85 average chemical fingerprint Tanimoto similarity score. Rimegepant (DB12457) showed the 2nd lowest binding energy of -11.43 kcal/mol. This drug is used as an oral calcitonin gene-related peptide (CGRP) receptor antagonist for the acute treatment of migraines in adults [79]. This compound shows a 0.90 average chemical fingerprints Tanimoto score compared with remdesivir. Rimegepant (DB12457) forms a hydrogen bond with D618 (2.7 Å), Y619 (2.9 Å), K621 (3.0 Å) (2.9 Å), E811 (2.7 Å), and C813 (3.5 Å) and hydrophobic interactions with D618 and D761 (Fig. 4) (Table s1). Irinotecan (DB00762) shows a 0.93 average chemical fingerprint Tanimoto similarity score, and this compound had − 11.40 kcal/mol binding energy by forming hydrophobic interactions with K593, L758, and D761 and hydrogen bonds with G590 (3.2 Å) and T591 (3.2 Å) (Fig. 4) (Table s1). This drug is used to treat rectal and colon metastatic carcinoma through its action as an antineoplastic enzyme inhibitor [80].

**Fig. 5:**
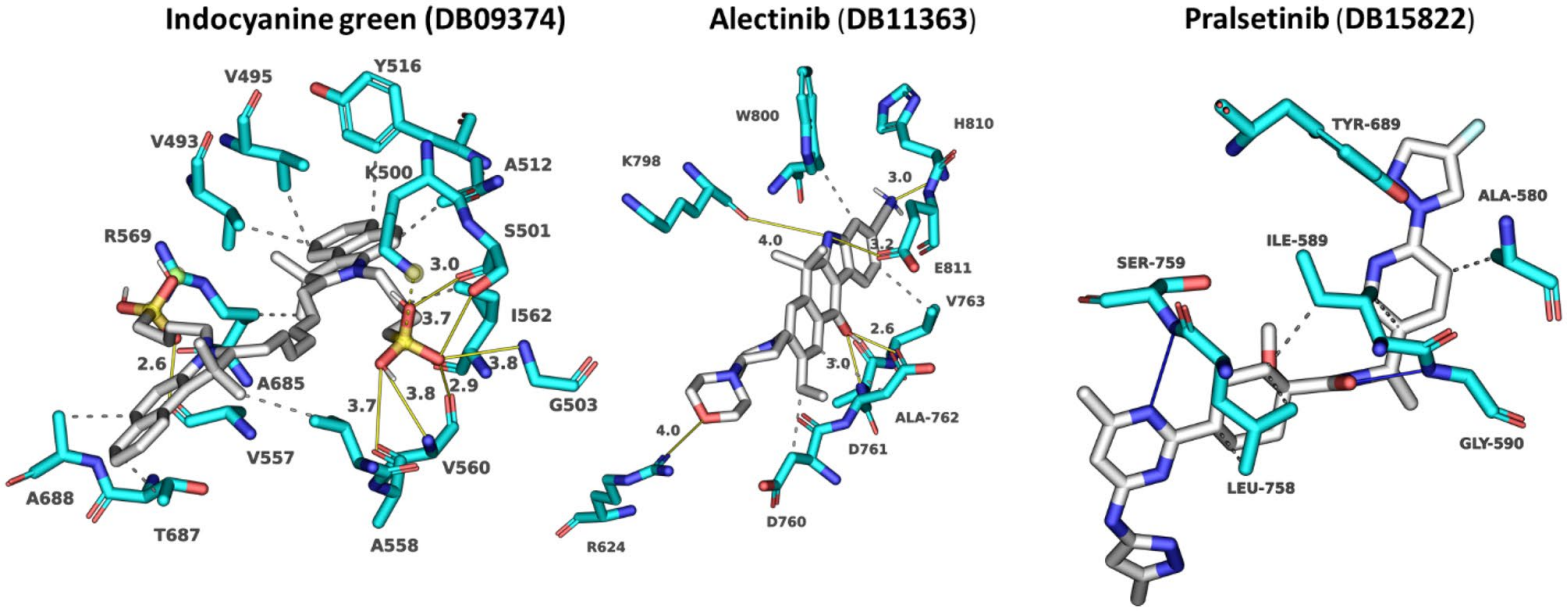
Modelled interactions between SARS-CoV-2 RdRp and indocyanine green, alectinib, and pralsetinib. Protein–ligand interactions formed between the screen hit compounds (grey) by hydrophobic interaction (grey dotted line), salt bridges (dotted yellow line), and H-bonds (solid yellow line) with numbered residues (cyan) in the binding tunnel of RdRp are shown

Indocyanine green (DB09374) is a water-soluble compound used as a diagnostic agent for cardiac output, hepatic function, liver blood flow, and ophthalmic angiography [81, 82]. This compound showed a binding energy of − 10.84 kcal/mol and a 0.82 average chemical fingerprint Tanimoto similarity score compared to remdesivir. The drug forms putative hydrophobic interactions with V493, V495, A512, Y516, V557, I562, R569, T687, and A688 and hydrogen bonds with S501 (3.0 Å) (3.7 Å), G503 (3.8 Å), A558 (3.7 Å), V560 (3.8 Å) (2.9 Å), and A685 (2.6 Å) (Fig. 5; Table s1). Alectinib (DB11363) is a second-generation oral kinase inhibitor used to inhibit anaplastic lymphoma kinase (ALK) tyrosine kinase activity specifically to treat metastatic non-small cell lung cancer [83]. This compound is proposed to form hydrogen bonds at R624 (4.0 Å), D761 (2.6 Å), A762 (3.0 Å), K798 (3.9 Å), H810 (3.0 Å), and E811 (3.2 Å) and hydrophobic interactions at D760, D761, V763, and W800 (Fig. 5; Table s1) with a binding energy of − 10.51 kcal/mol. When compared to remdesivir, it shows a Tanimoto similarity score of 0.83. Pralsetinib (DB15822) shows a 0.89 average chemical fingerprints Tanimoto similarity score and − 10.34 kcal/mol binding energy, forming hydrogen bonds with G590 (2.8 Å), Y689 (2.9 Å), and S759 (3.7 Å) and hydrophobic interactions with A580, I589, and L758 (Fig. 5; Table s1). This drug is an inhibitor of the tyrosine kinase activity of the RET receptor, used to treat metastatic RET-driven non-small cell lung cancer [84]. Although a phase 1/2 trial of this drug is still ongoing, this drug was granted accelerated FDA approval on 4 September 2020, to treat metastatic RET-fusion-positive non-small cell lung cancer under the brand name GAVRETO™ by Blueprint Medicines.

**Fig. 6:**
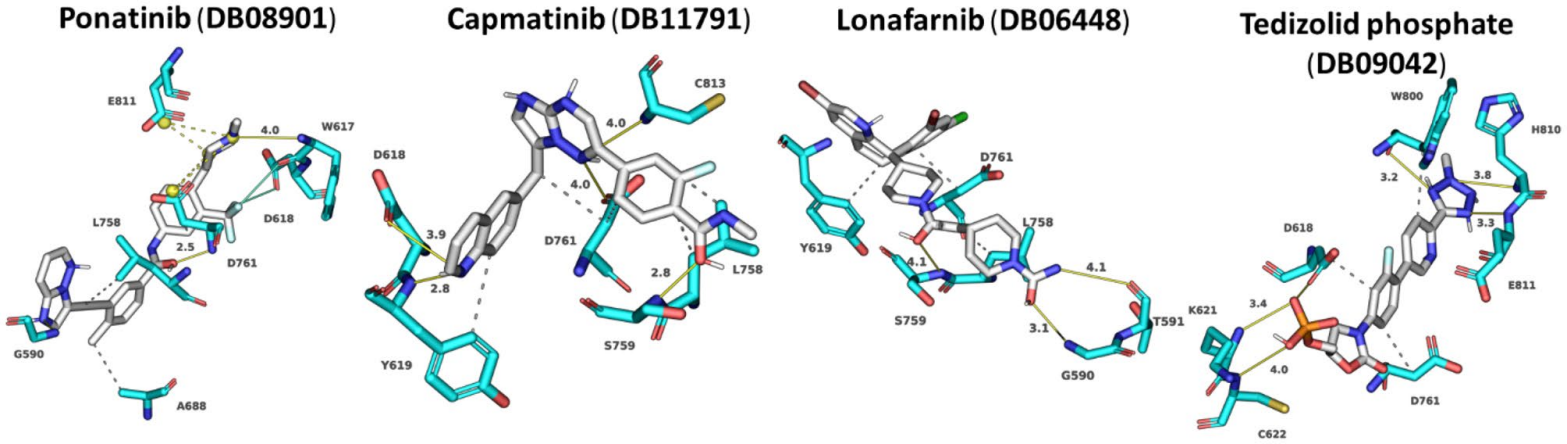
Modelled interactions between SARS-CoV-2 RdRp and ponatinib, capmatinib, lonafarnib, and tedizolid phosphate. Protein–ligand interactions formed between the screen hit compounds (grey) by hydrophobic interaction (grey dotted line), salt bridges (dotted yellow line), and H-bonds (solid yellow line) with numbered residues (cyan) in the binding tunnel of RdRp are shown

From the list of ten compounds, ponatinib (DB08901), capmatinib (DB11791), lonafarnib (DB06448), and tedizolid phosphate (DB09042) showed binding energies of − 10.11 kcal/mol, − 10.11 kcal/mol, − 10.09 kcal/mol, and − 10.06 kcal/mol, respectively. Docking predicts that ponatinib forms A688, L758, and D761 hydrophobic interactions and H-bonds with G590 (2.9 Å), W617 (4.0 Å), and D761 (2.5 Å) (Fig. 6; Table s1). This compound is a kinase inhibitor used to treat chronic myeloid leukaemia (CML) [85]. It shows a 0.82 average chemical fingerprints Tanimoto score compared with the reference compound. Capmatinib (DB11791) drug is also an FDA-approved kinase inhibitor, targeting c-Met receptor tyrosine to treat non-small cell lung cancer with Exon 14 skipping mutations [86]. This compound shows a 0.81 similarity score, and the docking predicts that it forms hydrophobic interactions with Y619, L758, and D761 and hydrogen bonds with D618 (3.9 Å), Y619 (2.8 Å), S759 (2.8 Å), D761 (4.0 Å), and C813 (4.0 Å) (Fig. 6; Table s1). Lonafarnib (DB06448) and tedizolid phosphate (DB09042) show Tanimoto scores of 0.82 and 0.84, respectively. DB06448 is a potent farnesyl transferase inhibitor prescribed to reduce mortality associated with Hutchinson-Gilford progeria syndrome (HGPS) [87]. Tedizolid phosphate is an oxazolidinone class antibiotic that inhibits bacterial protein synthesis and is proven effective in treating certain Gram-positive bacterial infections [88]. Residues predicted to be involved in hydrophobic and hydrogen bond interactions are shown in Table s1, which lists residues present within 5 Å of the selected FDA drug.

### Clustering

**Fig. 7:**
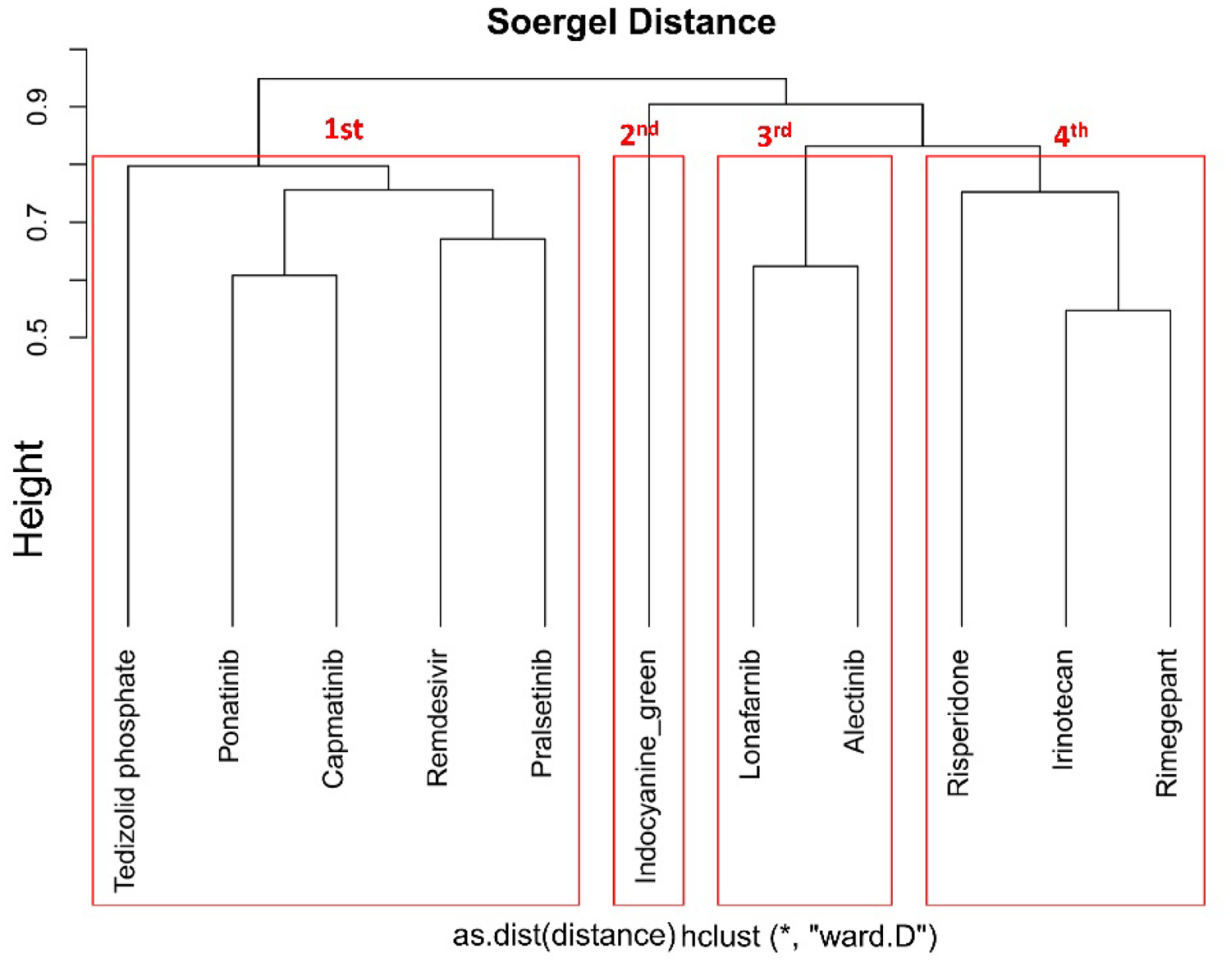
Clustering of putative inhibitors of SARS-CoV-2 RdRp. ChemBioServer clustering found four clusters based on hierarchical clustering. The first cluster shows five compounds, including the reference compound remdesivir

After docking, we performed clustering analysis for the selected ten hit compounds and the reference compound remdesivir, to re-rank them using the ChemBioServer tool. Four clusters were identified using this approach (Fig. 7). The top Cluster-1, which contains remdesivir, ponatinib (DB08901), tedizolid phosphate (DB09042), capmatinib (DB11791), and pralsetinib (DB15822) were used for MD simulation and binding-free energy calculation (Fig. 7).

The selected ten compounds were compared with the reference remdesivir drug to check the structural difference (Fig. s3). The ProFit Server compares the two structures and gives an RMSD fit score based on fitting the two structures. Risperidone (DB00734) shows the highest RMSD fit score (6.916), and the lowest RMSD fit score is 3.783, shown by irinotecan (DB00762). The RMSD fit scores for all 10 selected compounds are shown in Table 2.

**Table 2:** Predicted RMSD scores for fitting of putative RdRp inhibitors compared to reference compound

**Compound name**	**Fit RMSD score**	**Compound name**	**Fit RMSD score**
Risperidone (DB00734)	6.916	Pralsetinib (DB15822)	4.983
Rimegepant (DB12457)	4.712	Ponatinib (DB08901)	4.395
Irinotecan (DB00762)	3.783	Capmatinib (DB11791)	5.120
Indocyanine green (DB09374)	5.417	Lonafarnib (DB06448)	6.205
Alectinib (DB11363)	4.794	Tedizolid phosphate (DB09042)	4.366

### Molecular dynamic analysis

**Fig. 8:**
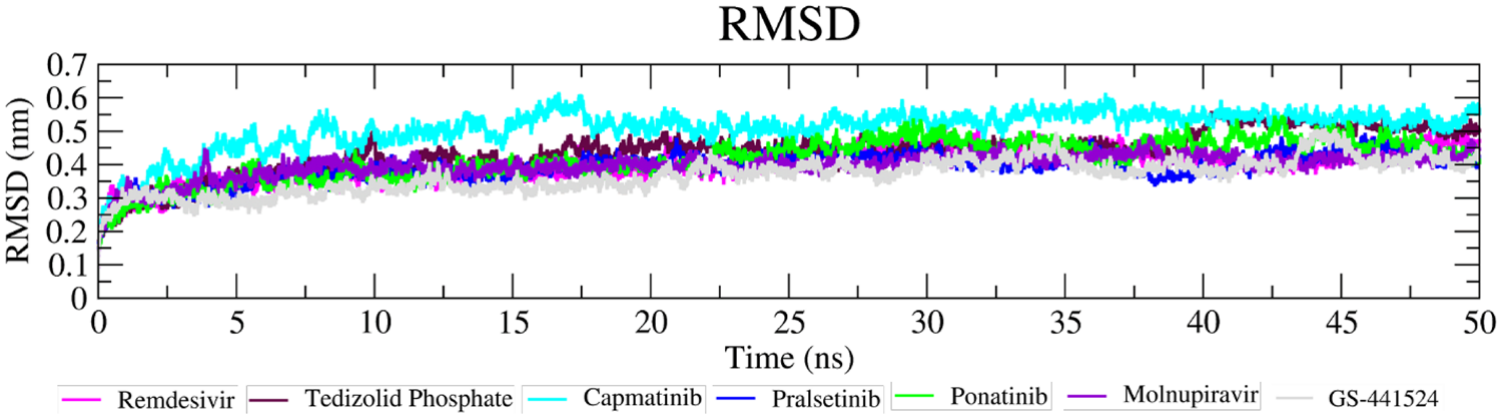
Molecular dynamic simulation of RdRp-ligand complexes. RMSD of the Cα backbone for selected protein–ligand complex compounds for 50-ns MD simulation

The four best FDA-approved compounds after the virtual screening and clustering were further investigated for their binding characteristics and dynamic behaviour. Along with these compounds, we include the active metabolite GS-441524. In a 50-ns MD simulation of protein–ligand complexes with two Zn^2+^ restraints, all systems achieved stability after 3 ns with RMSD value between 0.25 and 0.55 nm throughout the simulation. Capmatinib (Fig. 8: cyan) showed an increase in RMSD value from the start of simulation up to 17.5 ns, followed by a steady fluctuation between 0.45 and 0.55 nm up to end of 50-ns simulation. Remdesivir (Fig. 8: magenta) showed a steady RMSD value up to 35 ns followed by a slight decrease in the RMSD value between 0.4 and 0.5 nm throughout the 50-ns simulation (Fig. 8). In the case of a second reference compound molnupiravir (Fig. 8: purple) used for comparison shows a slow increase in RMSD occurs in the initial period up to 1 ns. It then shows a slight decrease in RMSD value before a slow increase in RMSD value throughout the simulation. The end simulation’s end shows the 0.4 nm RMSD value (Fig. 8: purple). Overall, remdesivir and molnupiravir show the same RMSD pattern, although molnupiravir shows a slightly higher RMSD value throughout the time course of the MD simulation.

Tedizolid phosphate (Fig. 8: maroon) shows a greater RMSD value after 38 ns, between 4.5 and 5.5 nm RMSD (Fig. 8). Pralsetinib unusually showed stable RMSD values throughout the simulation but with lower RMSD compared with remdesivir, tedizolid phosphate, and capmatinib of between 3.0 and 4.5 nm (Fig. 8). In the case of ponatinib, the same pattern of fluctuation is seen up to 27-ns simulation; after that, a slight increase in the RMSD is evident in comparison to the other hit compounds (Fig. 8: green) up to the end of the simulation. Compared to remdesivir, it shows a lower RMSD value at the start of the simulation, but after 10 ns, its shows a higher RMSD compared to the reference compound. Active metabolite GS-441524 (Fig. 8: grey) shows an increase in RMSD value at the start of simulation up to 1 ns, before we observe a steady increase in the RMSD value up to the end of the 50-ns MD simulation. Overall, the RMSD calculations suggest that binding of the identified compounds significantly stabilized the RdRp-ligand complex structure in a way similar to that achieved with the reference compounds known to be effective RdRp inhibitors.

Further analysis was performed by calculating RMS fluctuations to provide information about the effects of the putative inhibitors on motions in RdRp. RMSF plots were drawn as a function of residue position for the 50-ns simulation. RMSF value was calculated by plotting motion (in nm) versus residue (Fig. s4). Capmatinib and ponatinib showed higher RMSF values compared to remdesivir, tedizolid phosphate, and pralsetinib, consistent with conformational shifts associated with binding these compounds. Fluctuation was induced by capmatinib at RdRp residue regions 220–230, 250–260, 375–400, 450–500, 530–650, and 760–775 amino acids when compared with remdesivir, tedizolid phosphate, and pralsetinib. In the case of ponatinib, a higher RMSF value is seen in the first (N-terminal region) 175 amino acids and a higher RMSF value between 875 and 910 amino acids (Fig. s4). GS-441524 compound showed the same pattern of RMSF values across the simulation as shown by remdesivir but with overall lower RMSF values than for the pro-drug. In general, residues in the binding tunnel (residues 585–830) showed lower RMSF values when it is bound to remdesivir and the selected candidate compounds, suggesting stabilization of the structure. Gyration (Rg) analysis provides a measure of the protein–ligand complex compactness. In the initial simulation stages, Rg values for RdRp with either reference or hit compounds were decreased. However, after 50-ns simulation, Rg values for reference and hit compounds were consistently between 3.1 and 3.3 nm (Fig. s5), consistent with comparable compactness in the structure of the complexes. Comparing compactness with the known remdesivir complex shows a lower Rg value at the initial simulation than the known remdesivir compound. Only ponatinib and the reference compound molnupiravir showed some evidence of decreased compactness, with Rg values between 3.2 and 3.3 nm (Fig. s5).

**Fig. 9:**
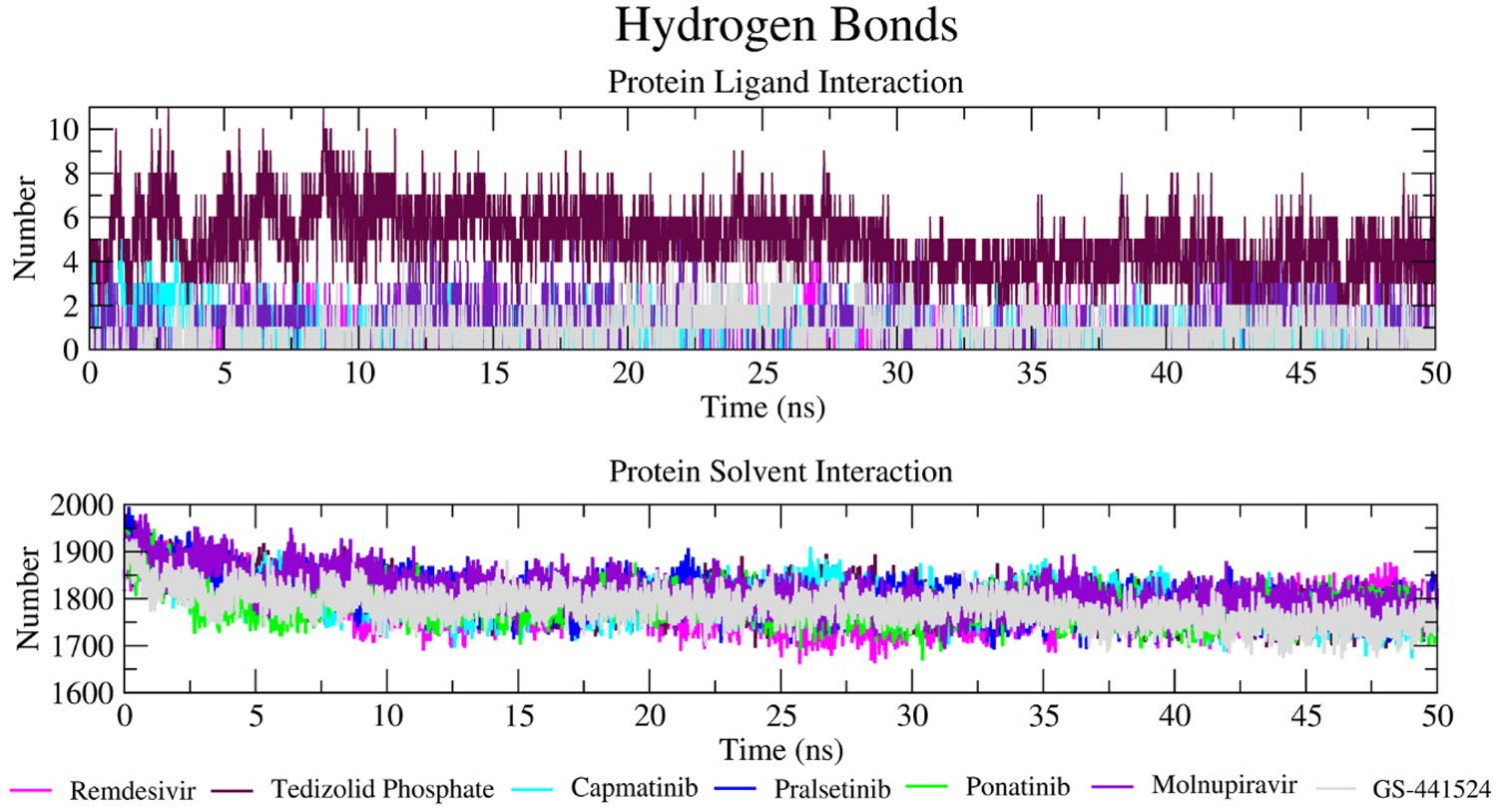
Trajectory of hydrogen bond interactions between RdRp and selected hit compounds during MD simulation. **A** Total number of hydrogen bonds formed between RdRp-ligand complex. **B** Total number of hydrogen bond interactions between protein and solvent

Hydrogen bond formation between protein and ligand and protein and solvent throughout the simulation was also analysed (Fig. 9). Hydrogen bond formation is of course critical in stabilizing the protein–ligand complex and is responsible for drug specificity, metabolization, and adsorption in the body. Hydrogen bond formation between RdRp and tedizolid phosphate involved more hydrogen bonds than was seen with reference compounds, with more than ten hydrogen bonds forming between 3–6- and 8–9-ns simulation (Fig. 9A). In the case of reference compounds remdesivir and GS-441524 compound, fewer than five hydrogen bonds are formed. Capmatinib showed no more than two hydrogen bonds throughout the simulation. Pralsetinib is predicted to make more than three hydrogen bonds between 5- and 22-ns simulation but after that makes fewer than three hydrogen bonds up to the end of 50-ns simulation (Fig. 9A). Hydrogen bond formation between protein and solvent throughout the 50-ns simulation (Fig. 9B) was constant throughout the simulation for all RdRp-ligand complexes, with between 1600 and 2000 hydrogen bonds (Fig. 9B). All hit compounds and reference compounds showed that hydrogen bond formation correlated positively with simulation time. Through this protein–ligand and protein-solvent hydrogen bond interaction, we conclude that reference compounds and hit compounds interact with RdRp effectively and tightly through this hydrogen bond interaction.

### Principle component analysis (PCA)

The eigenvectors, eigenvalues, and their projection were calculated using the essential dynamics methods to perform PCA. Through this method, we analysed the motion of the protein–ligand complex during simulation. These eigenvectors determine the overall motion of the particular protein. The protein–ligand complexes can be explained by 2D projection plot generation in PCA. We selected the first two principal components, PC1 and PC2, to predict the significant motions. PCA for hit and reference compounds showed stable motion, although capmatinib did show more moments on PC1 and PC2. Reference compound remdesivir showed a more positive PC value (Fig. s6). Tedizolid phosphate and pralsetinib showed the same moments on the PC1 and PC2 axis, indicating stable clusters.

### Free energy calculation

We used trajectory files in MMPBSA for both the reference and screen hit compounds for free energy calculation. Through this, we analysed the interactions by energy point and identified the configuration of the compound in the complex. We used the 100 frames from the 50-ns trajectory files to calculate the free energy. After MD simulation for remdesivir, the free energy was − 835.634 kJ/mol and for GS-441524 was − 665.436 kJ/mol. The other three hit compounds, tedizolid phosphate, capmatinib, and pralsetinib, showed − 51.972 kJ/mol, − 67.057 kJ/mol, and − 130.474 kJ/mol, respectively (Table s2). Ponatinib shows the highest binding energy and molnupiravir the lowest. Van der Waal, electrostatic, polar solvation energy, and SASA energy values are also shown in Table s2.

## Conclusion

In this study, a virtual screen has identified and characterized the binding of a series of FDA-approved compounds as potential new inhibitors of SARS-CoV-2 RdRp. RdRp is essential for RNA synthesis in all positive-strand RNA virus replication in coronavirus. From an initial 2509 FDA-approved compounds, a cluster of four compounds were identified that could be modelled as stable ligands for RdRp that bound with similar characteristics as previously identified inhibitors remdesivir and molnupiravir and the remdesivir metabolite GS-441524. Based on calculated binding energies and measures of conformational movement in the protein–ligand complexes, these compounds bind strongly to the template binding/active site region of the polymerase via a range of hydrophobic, dipole, and ionic interactions to form stable complexes. On that basis, pralsetinib (DB15822), ponatinib (DB08901), capmatinib (DB11791), and tedizolid phosphate (DB09042) may have sufficient potential for treatment of SARS-CoV-2 infection to be worth following up with evaluation of their effects in biological models.

## Supplementary Information

Below is the link to the electronic supplementary material.

Supplementary file1 (DOCX 1728 KB)

## Abbreviations

RNA: Ribonucleic acid
FDA: Food Drug and Administration
WHO: World Health Organization
SARS: Severe acute respiratory syndrome
MERS: Middle East respiratory syndrome
β-CoV: β-Coronavirus
NSP: Non-structural proteins
MPRO: Main protease
PLPRO: Papain-like protease
3CLPRO: Chymotrypsin-like protease
RdRp: RNA-dependent RNA polymerase
2D: 2 Dimensional
3D: 3 Dimensional
SDF: Structure data file
Tc: Tanimoto coefficient
PDB: Protein Data Bank
Å: Angstrom
ADT: AutoDock tools
PLIP: Protein-ligand interaction profile
RMSD: Root mean square deviation
MD simulation: Molecular dynamic simulation
ns: Nanosecond
SPC: Simple point charge
NVT: Canonical ensemble, amount of substance (N), volume (V), and temperature (T)
NPT: Isothermal–isobaric ensemble, amount of substance (N), pressure (P), and temperature (T)
RMSF: Root mean square fluctuation
Rg: Radius of gyration
MM-PBSA: Mechanics energies combined with Poisson-Boltzmann
BFE: Binding-free energy
CF: Chemical fingerprint
Tan: Tanimoto
BE: Binding energies
