# Supplementary material for: Identification of FDA-approved drugs against SARS-CoV-2 RNA-dependent RNA polymerase (RdRp) through computational virtual screening
Source: Struct Chem. 2022 Nov 25;34(3):1005–19. doi: 10.1007/s11224-022-02072-1 (PMC9702953; doi:10.1007/s11224-022-02072-1)
Supplement: Supplementary file 1 — Supplementary file1 (DOCX 1728 KB) [file 11224_2022_2072_MOESM1_ESM.docx]

***Supplementary Information***

**Identification of FDA-approved drugs against SARS-Co-2 RNA-dependent RNA polymerase (RdRp) through computational virtual screening.**

Dhananjay Jade^1#^, Areej Alzahrani^2^, William Critchley^2^, Sreenivasan Ponnambalam^2^, and Michael A. Harrison^1*^

^1^School of Biomedical Sciences, University of Leeds, UK.

^2^ School of Molecular & Cellular Biology, University of Leeds, UK.

*Correspondence Author: m.a.harrison@leeds.ac.uk

^#^Communication address: bsddj@leeds.ac.uk

**Figures**


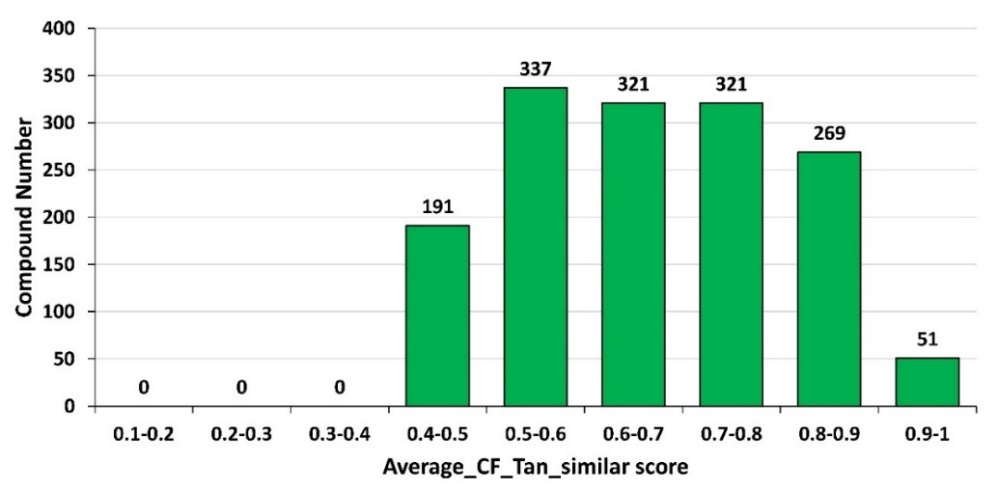


**Fig s1.** The graph represents the calculated Average Chemical Fingerprint (CF) Tanimoto (Tan) similar score range for selected compounds.


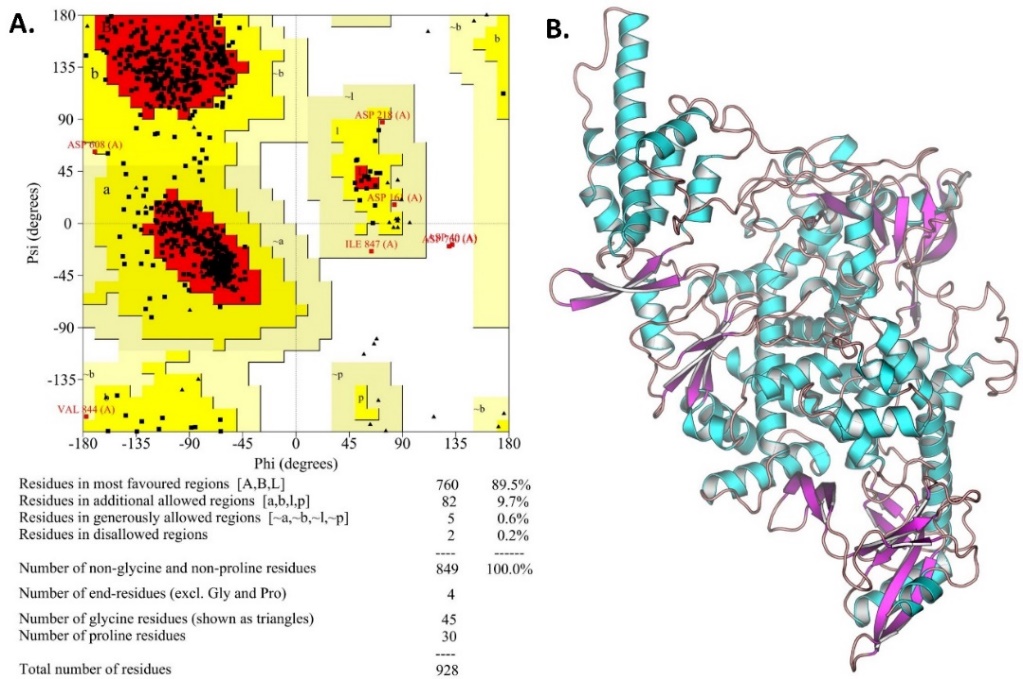


**Fig s2.** Analysis of protein structure after adding missing residues **A.** Ramachandran plot analysis after adding missing residues shows 89.5% of residues are in a favorable region. **B.** RdRp 3D protein structure after adding missing residues, magentas color shows the beta-sheets, and cyan color shows the helix structure.


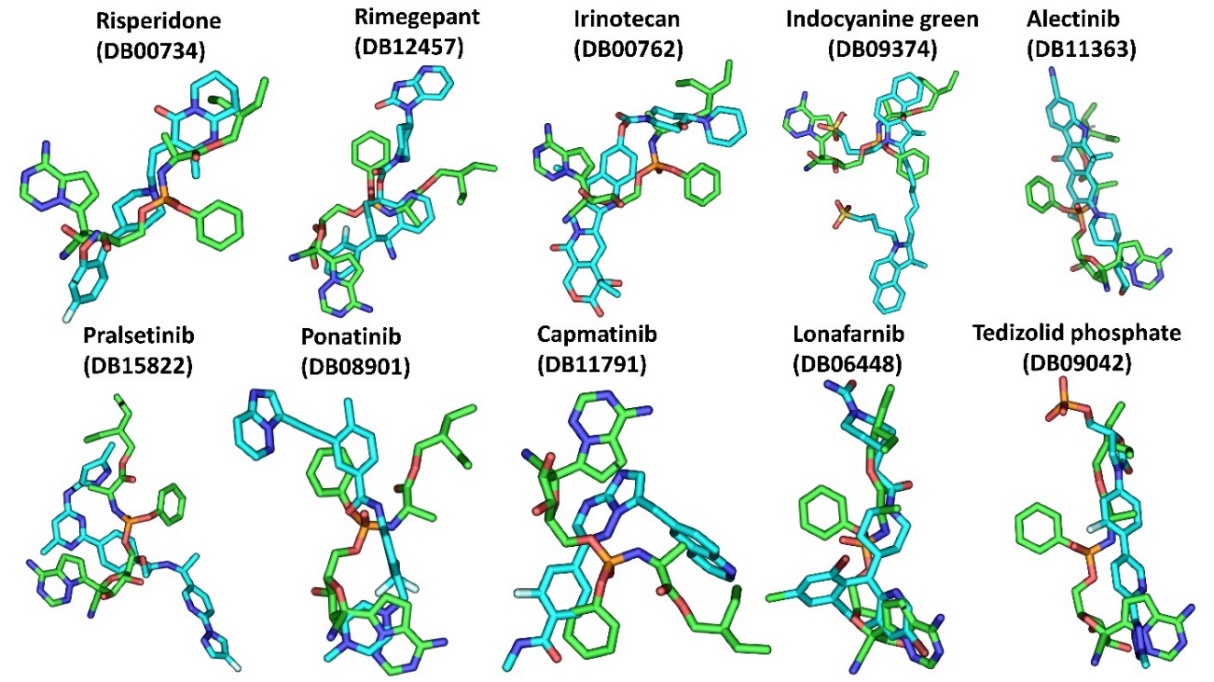


**Fig s3.** Variations for virtual hit compounds against reference Remdesivir compound: aligned comparison shown for selected ten compounds. Selected hit compounds are shown in cyan color and the reference Remdesivir compound is shown in green color.


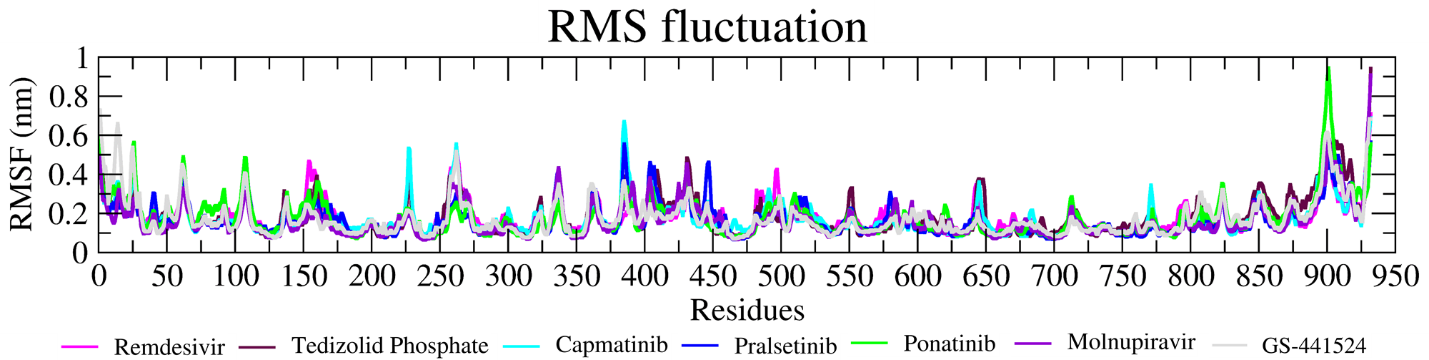


**Fig s4.** Molecular dynamic simulation of RdRp-ligand complex. RMSF for selected protein-ligand complex compounds for 50 ns MD simulation.


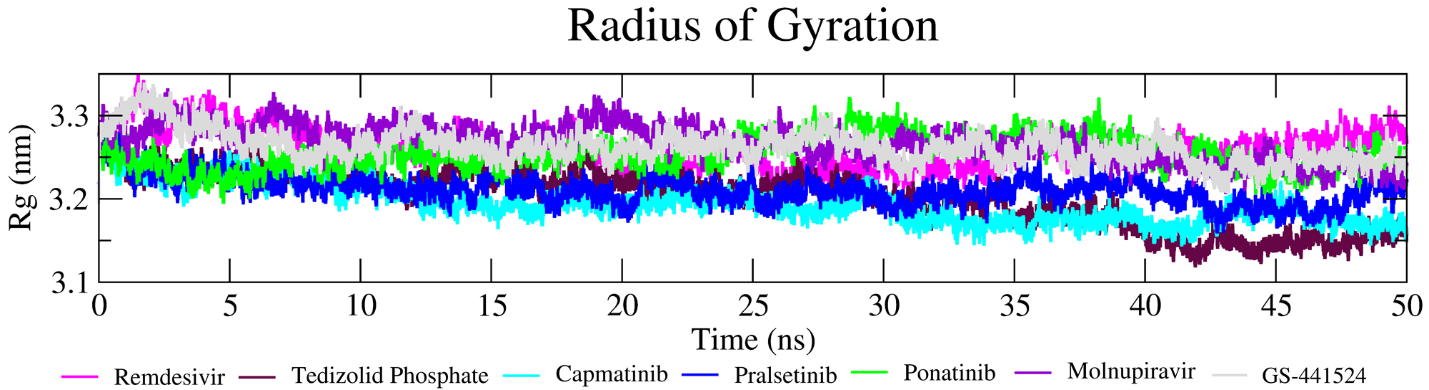


**Fig s5.** Molecular dynamic simulation of RdRp-ligand complex. Rg for selected protein-ligand complex compounds for 50 ns MD simulation.


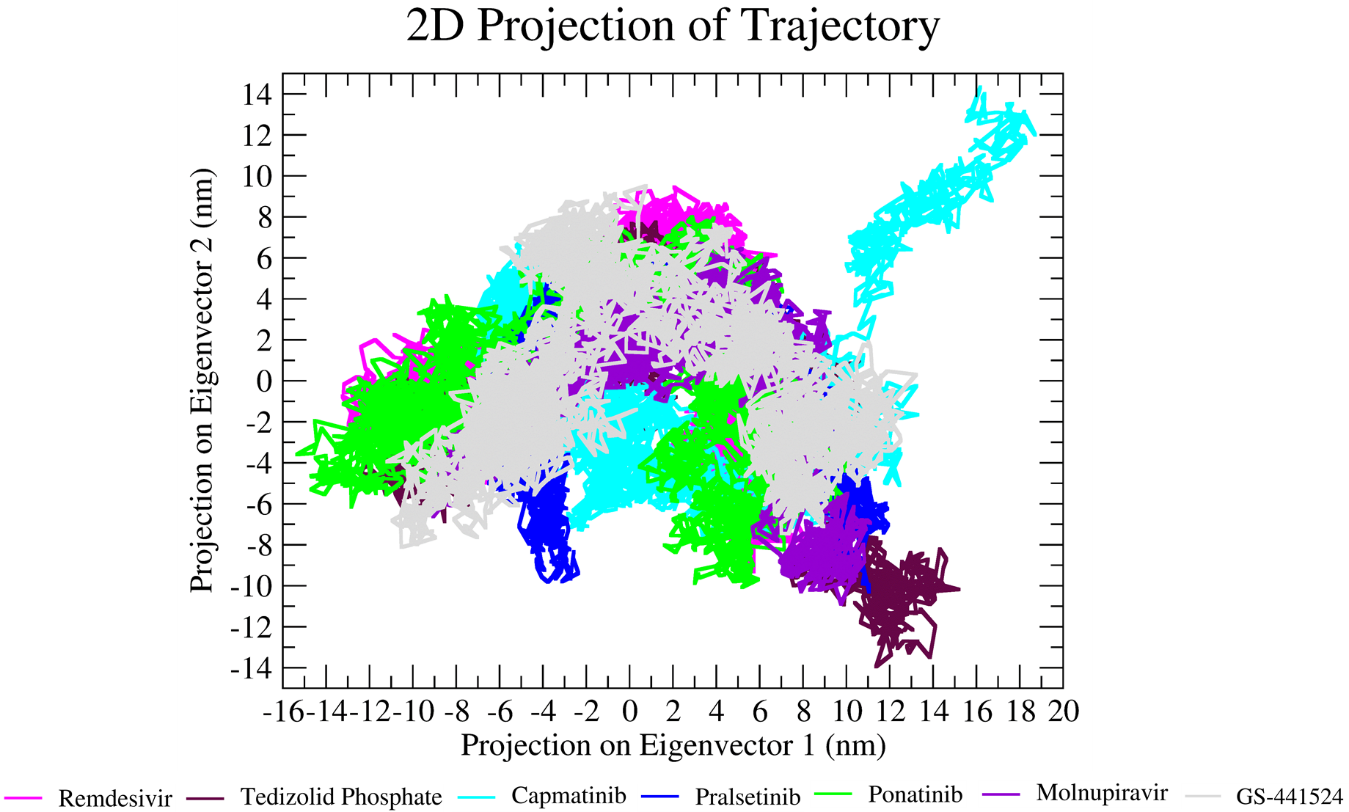


**Fig s6.** Principle component analysis of RdRp-ligand complex: Projection of Cα atoms of the motion of RdRp-ligand complex in phase space along the first two eigenvectors (PC1 and PC2) of RdRp-ligand complex.

**Table**

**Tab s1.** The selected top compound shows amino acids involved in interaction within 5Å of Compounds.

| **Compound Name** | **Amino Acid within 5Å of Compounds** |
| --- | --- |
| Remdesivir | A580, I589, G590, T591, S592, K593, F594, T687, A688, L758, S759, D760, D761, C813, S814, Q815 |
| Risperidone (DB00734) | L614, M615, G616, W617, D618, Y619, P620, K621, C622, D623, D760, D761, A762, V763, V764, K798, W800, T801, E802, P809, H810, E811, F812, C813, S814 |
| Rimegepant (DB12457) | D452, A554, G616, W617, D618, Y619, P620, K621, C622, D623, R624, D760, D761, A762, V763, K798, C799, W800, E811, F812, C813, S814 |
| Irinotecan (DB00762) | V588, I589, G590, T591, S592, K593, G616, W617, D618, Y619, C622, A688, L758, S759, D760, D761, A762, V763, K798, W800, E811, F812, C813, S814 |
| Indocyanine green (DB09374) | V495, K500, A512, V560, I562, T565, R569, L576, I589, G590, T591, S682, G683, D684, A685, T686, T687, A688, Y689, N691, L758, S759, D760, D761 |
| Alectinib (DB11363) | L614, M615, G616, W617, D618, Y619, P620, K621, C622, D623, R624, S759, D760, D761, A762, V763, K798, C799, W800, T801, E802, P809, H810, E811, F812 |
| Pralsetinib (DB15822) | R569, Q573, L576, K577, A580, V588, I589, G590, T591, A688, Y689, L758, S759, D760, D761, E811, C813, S814 |
| Ponatinib (DB08901) | V588, I589, G590, T591, S592, K593, G616, W617, D618, Y619, C622, A688, L758, S759, D760, D761, A762, V763, K798, W800, E811, F812, C813, S814 |
| Capmatinib (DB11791) | I589, G590, W617, D618, Y619, P620, C622, L758, S759, D760, D761, A762, E811, C813, S814, R836 |
| Lonafarnib (DB06448) | V588, I589, G590, T591, K593, G616, W617, D618, Y619, K621, C622, L758, S759, D760, D761, A762, E811, C813, S814 |
| Tedizolid phosphate (DB09042) | L614, M615, G616, W617, D618, Y619, P620, K621, C622, D760, D761, A762, V763, K798, W800, T801, E802, P809, H810, E811, F812 |
| Molnupiravir (DB15661) | V493, I494, V495, N496, N497, L498, K500, S501, A502, G503, A512, R513, Y515, Y516, M519, A558, G559, V560, S561, I562, T565 M566, R569, G683, A685 |

**Tab s2.** Binding free energy calculated for RdRp-ligand complex. Van der Waal energy, Electrostatic energy, Polar solvation energy, SASA energy, and Binding energy in kJ/mol.

| ZINC ID | Van der Waal energy (kJ/mol) | Electrostatic energy (kJ/mol) | Polar solvation energy (kJ/mol) | SASA energy (kJ/mol) | Binding energy (kJ/mol) |
| --- | --- | --- | --- | --- | --- |
| Remdesivir | -41.692 | -1083.604 | 297.622 | -7.960 | -835.634 |
| Tedizolid phosphate | -75.417 | -17.683 | 50.173 | -9.045 | -51.972 |
| Capmatinib | -136.852 | -89.324 | 175.335 | -16.216 | -67.057 |
| Pralsetinib | -163.133 | -5.825 | 53.855 | -15.371 | -130.474 |
| Ponatinib | -84.907 | -1751.531 | 807.339 | -13.023 | -1042.122 |
| Molnupiravir | -107.752 | 4.448 | 114.798 | -11.906 | -0.412 |
| GS-441524 | -41.179 | -842.856 | 224.314 | -5.715 | -665.436 |
